# Neuropsychological assessment protocol in an ongoing randomized controlled trial on posterior subthalamic area vs. ventral intermediate nucleus deep brain stimulation for essential tremor

**DOI:** 10.3389/fneur.2023.1222592

**Published:** 2023-10-30

**Authors:** Lucía Triguero-Cueva, Bartolomé Marín-Romero, Carlos Javier Madrid-Navarro, María José Pérez-Navarro, Benjamin Iáñez-Velasco, Adolfo Mínguez-Castellanos, Majed Jouma Katati, Francisco Escamilla-Sevilla

**Affiliations:** ^1^Department of Neurology Hospital Universitario Virgen de las Nieves, Granada, Spain; ^2^Instituto de Investigación Biosanitaria ibs. GRANADA, Granada, Spain; ^3^Department of Neuropsychology Hospital Universitario Virgen de las Nieves, Granada, Spain; ^4^Department of Neurosurgery Hospital Universitario Virgen de las Nieves, Granada, Spain

**Keywords:** cognitive-affective impairment, deep brain stimulation, essential tremor, neuropsychological assessment, posterior subthalamic area, ventral intermediate nucleus, surgical therapies

## Abstract

**Objective:**

Patients with essential tremor (ET) may experience cognitive-affective impairment. Deep brain stimulation (DBS) of different targets, such as the ventral intermediate nucleus (VIM) of the thalamus or the posterior subthalamic area (PSA), has been shown to be beneficial for refractory ET. However, there is little evidence regarding the possible neuropsychological effects of PSA-DBS on patients with ET, and there are few studies comparing it with VIM-DBS in this population.

In this study, we aim to present the evaluation protocol and neuropsychological battery as used in an ongoing trial of DBS for ET comparing the already mentioned targets.

**Methods:**

As part of a randomized, double-blind, crossover clinical trial comparing the effectiveness and safety of PSA-DBS vs. VIM-DBS, 11 patients with refractory ET will undergo a multi-domain neuropsychological battery assessment. This will include a pre−/post-implantation assessment (3 months after the stimulation of each target and 6 months after an open stage of DBS on the most optimal target).

**Conclusion:**

Evidence on the neuropsychological effects of DBS in patients with refractory ET is very scarce, particularly in lesser-explored targets such as PSA. This study could contribute significantly in this field, particularly on pre-procedure safety analysis for tailored patient/technique selection, and to complete the safety analysis of the procedure. Moreover, if proven useful, this proposed neuropsychological assessment protocol could be extensible to other surgical therapies for ET.

## 1. Introduction

Essential tremor (ET) is the most common movement disorder (1). Due to its association with ageing, its prevalence has increased in recent decades, reaching 2.3–14.3% in people over 60 (2). Moreover, recent research has shown that ET, rather than being a monosymptomatic disorder, may be associated with other subtle neurological manifestations. As such, patients may present with cerebellar dysfunction (ataxia, dysarthria, bradykinesia, and ocular movement anomalies) as well as cognitive impairment and mood disorders. These presentations have been denominated “essential tremor plus” in the International Parkinson and Movement Disorder Society classification (3).

Although cognitive impairment in ET is usually slight, it may affect multiple domains such as attention, concentration, executive function, memory, language, visuospatial function, and processing speed (Table 1). In addition, depression and anxiety are the most common neuropsychiatric manifestations (4).

**Table 1:** Affected cognitive domains in patients with essential tremor.

Attention Concentration Executive functions Working memory Cognitive inhibition Cognitive flexibility Memory Short-term memory Delayed memory Language Verbal fluency Processing speed Visuoperceptual abilities* Mood Depression Anxiety
**Cognitive-affective changes VIM-DBS**
May improve Visuoperceptual abilities Attention Word recognition skills Anxiety Depression May deteriorate Verbal fluency
Cognitive-affective changes PSA-DBS*
May deteriorate Verbal fluency

*Not consistently affected/not extensively evaluated.

Depending on the severity of the symptoms, ET can limit daily activities and affect quality of life (5). Medical treatment is the first option, but this is often unsatisfactory or limited by side effects (6). Deep brain stimulation (DBS) may be an alternative to medication for patients with incapacitating and refractory ET (7), and different anatomical areas have shown potential as surgical targets.

The ventral intermediate nucleus (VIM) of the thalamus is currently the target of choice (8); however, the possible appearance of a tolerance phenomenon for tremor control (9) or long-term side effects such as gait ataxia (10) and dysarthria (11) may limit the benefits of VIM-DBS. In terms of changes in the cognitive-affective sphere after VIM-DBS, the evidence has shown improvement in visuoperceptual abilities, visual attention, and word recognition skills, as well as in anxiety, depression, and quality of life. Conversely, verbal fluency may deteriorate after surgery (12–16), with pre-surgical reduction (12) and high-frequency stimulation (that which best controls the tremor) (15) being predictive factors.

Recently, the posterior subthalamic area (PSA) has reappeared as a potential alternative target (17, 18). The PSA contains fibres from the dentato-rubro-thalamic tract (DRTT) that are thought to play a crucial role in tremor physiopathology (19). Located ventral to the VIM, it is surrounded by several anatomical landmarks such as the zona incerta (ZI), the medial lemniscus, the fields of Forel (H1 and H2), the subthalamic nucleus, the red nucleus, and the pre lemniscal radiation (20). Currently, there are little data on the potential neuropsychological effects of DBS in this area. However, a decrease in verbal fluency has been described following ZI-DBS (21, 22), without significant changes in other cognitive functions or differences between uni- and bilateral interventions (21).

Regarding the efficacy of DBS on both targets, a controlled randomized clinical trial showed a trend toward better tremor control with lower stimulation amplitudes with PSA-DBS (18). Nonetheless, speech worsened in both groups (somewhat more after VIM-DBS, but not significantly) when measured with a visual analogue scale. Depression and quality of life were assessed in this study, but data on cognitive functions were not available.

Regarding other techniques, ablative functional surgery became obsolete after DBS was developed, but more recently, the use of unilateral thalamotomy using high-intensity focussed ultrasound (HIFU) has been approved. Dysarthria and ataxia are the most frequent adverse effects (23). A patient who received bilateral VIM thalamotomy did not experience any changes in the neuropsychological assessment (24). On the other hand, 5 patients of a series of 21 that underwent PSA-HIFU (3 treated bilaterally) (25) showed no evidence of changes in cognitive function or anxiety/depression scores (26).

As we see in the literature, there is not a widely accepted protocol for the neuropsychological assessment of patients with ET, unlike in other movement disorders. It would be beneficial to develop and implement a standardized assessment protocol to improve homogeneity and allow comparison between clinical studies on ET surgical treatment.

Our objective was to present a neuropsychological battery designed *ad hoc* as part of a controlled randomized clinical trial comparing PSA-DBS with VIM-DBS in patients with ET.

## 2. Methods and analysis

### 2.1. Study design

This neuropsychological study protocol will be applied as part of a randomized, crossover, double-blind clinical trial designed to evaluate the efficacy, effectiveness, and safety of PSA-DBS vs. VIM-DBS in ET as a secondary objective. The study has been approved by the Provincial Ethics Committee of Granada. Participants will undergo bilateral DBS using octopolar electrodes (Vercise®, Boston Scientific) at the level of the VIM (proximal contacts) and PSA (distal contacts). The stimulation sequence (PSA-VIM vs. VIM-PSA) will be randomized by random assignment. Neuropsychological assessments will be carried out in a blinded manner by the same neuropsychologist. Neuroimaging will include DRTT tractography to assess its relationship with the active contacts by calculating the volume of tissue activated (VTA) and the effects obtained. The study is already underway and is expected to last 3 years (completion date is expected in mid-2024).

### 2.2. Participants

The study will include a total of 11 patients with refractory ET, assessed and diagnosed by two expert neurologists as candidates for bilateral DBS treatment. The patients will meet the requirements to participate in the clinical trial (Table 2) and agree to participate in it. All participating subjects must provide their signed informed consent form. In addition to the neuropsychological examination, the evaluation of participants will include a detailed clinical history, the Fahn–Tolosa–Marin tremor rating scale (*FTM-TRS*), a neurological examination, and a preoperative 3 Tesla brain MRI, including diffusion tensor imaging (dTi) for tractography. The sample size for the original clinical trial was calculated through a hypothesis of non-inferiority, considering that the effect of the experimental technique (PSA-DBS) is no less than the standard technique (VIM-DBS) in tremor control (first endpoint: score on the FTM-TRS). This was calculated with Ene 3.0 software.^1^

**Table 2:** Inclusion and exclusion criteria for the clinical trial.

**Inclusion criteria**	Confirmed diagnosis of bilateral ET or ET-plus according to the Movement Disorders Society criteria. Refractoriness to medication: at least two attempts at medical treatment with at least two groups of different medication (fundamentally propranolol and primidone), which were ineffective (insufficient tremor control or adverse effects). Subjects of both sexes, older than 18 years old. Sufficient competence to collaborate and comply with the study protocols. - Ability to provide informed consent.
**Exclusion criteria**	Clinically relevant cognitive decline which may interfere with the study. Clinically relevant active psychiatric disorder. Contraindication for general surgery or bilateral DBS. Unsuitable electrode location according to neuroimaging. Participation in other interventional study. Cerebral atrophy (width of the third ventricle >10 mm) or other anatomical anomalies that would interfere with optimal stereotactic localization.

### 2.3. Study stages and collecting the neuropsychological data

**Figure 1:**
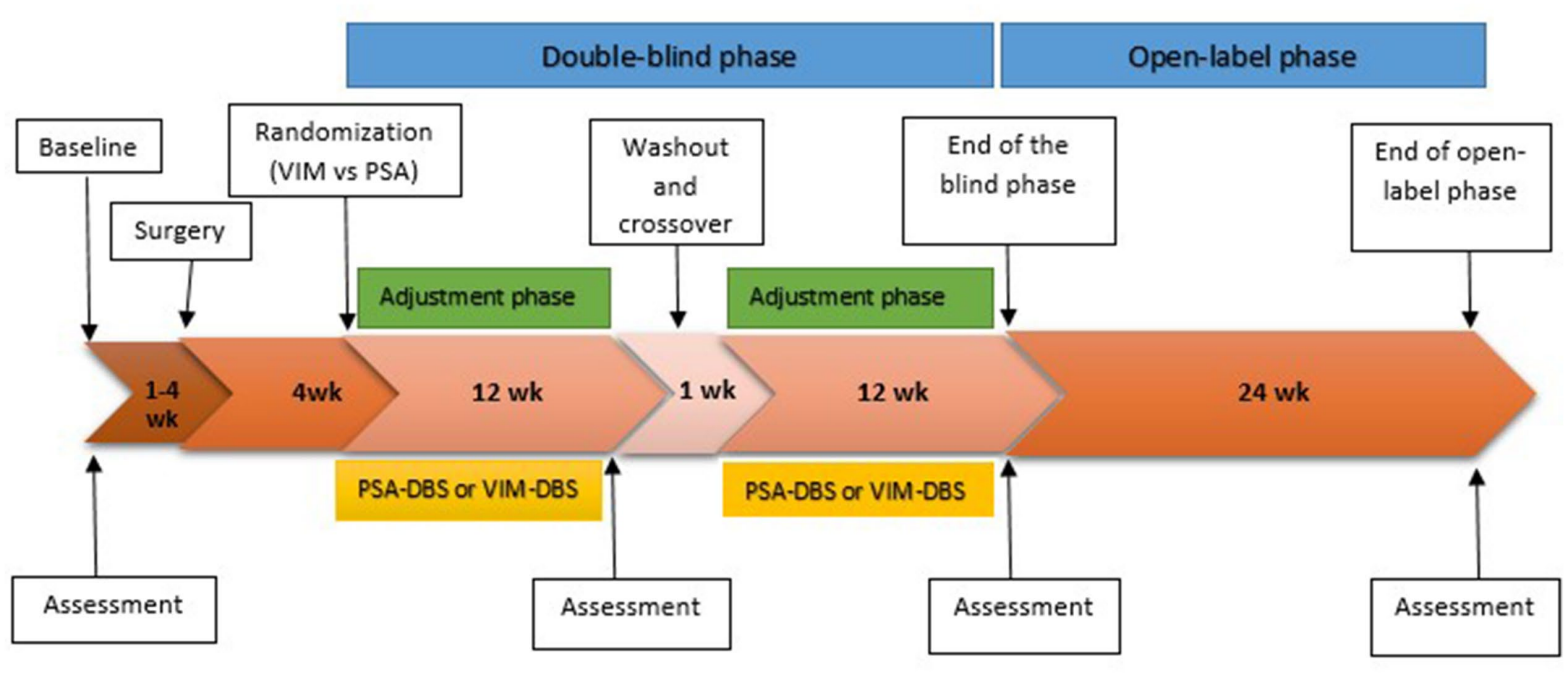
Study design. The downward-pointing arrows indicate study time points, and the upward-pointing arrows indicate assessments at those time points as per protocol.

All stages of the assessments will be carried out by the same neuropsychologist, blinded regarding the target, contacts, and stimulation parameters. Figure 1 outlines the workflow of the study (downward-pointing arrows indicate study time points, and upward-pointing arrows indicate assessments at those time points as per protocol).

Four assessments will be carried out: a pre-surgical assessment (visit 1 or baseline), a post-surgical assessment 3 months after the programming of the first randomized target (visit 2), and lastly, a post-surgical assessment 3 months after the programming of the second stimulation target (visit 3). The study will also include a final assessment following an open stage of 6-month duration, with DBS as the best target for the patient.

The baseline assessment will include a structured initial interview where the relevant socio-demographic and clinical data will be collected. All the assessments will include a neuropsychological evaluation and quality of life assessment. The subjective clinical impressions of the evaluator and subject will also be recorded. The rest of the clinical information, including tremor assessment, will be carried out in parallel, always by the same blinded neurologist, and recorded in the patient’s clinical history.

### 2.4. Cognitive-affective assessment: proposed neuropsychological assessment battery

#### 2.4.1. Interview

Relevant clinical and socio-demographic data, including age, sex, education, and profession, as well as the personal and family background of comorbid cognitive-behavioral impairments and active treatments that may have an influence on the neuropsychological assessment, will be collected from all the participants at the baseline visit through a structured interview.

The health characteristics related to the neurological history of the ET will also be collected, including the duration of the condition and age of onset.

#### 2.4.2. Cognitive assessment

Cognitive assessment includes several standardised tests extracted from widely used, validated neuropsychological batteries. A thorough literature review was performed to design the protocol by selecting the tests that would allow to specifically target the profile and domains affected in ET (4), thus achieving a comprehensive *ad hoc* cognitive examination for this pathology (tests and domains are listed in Table 3).

**Table 3:** Neuropsychological assessment protocol for candidates for ET treatment by DBS.

**Overall cognitive assessment**	Dementia Rating Scale 2
**Executive functions**	Wisconsin card sorting test Semantic and phonetic verbal fluency Stroop word-color test WAIS-IV working memory index Processing speed index
**Attention**	Trail making test A and B
**Memory and Language**	Complutense verbal learning test Spain Logical memory I and II and Weschler Memory scale IV Boston naming test
**Visuoperceptual and visuoconstructive**	Judgement line orientation test Clock-drawing Test WAIS-IV Block-design subtest
**Quality of life and subjective complaints**	Quality of life of essential tremor (QUEST). EuroQol-5D Prefrontal symptoms inventory (PSI)
**Psychopathology**	Beck’s depression inventory II

#### 2.4.3. Overall cognitive assessment

The Dementia Rating Scale-2 (DRS-2) is included in baseline screening since ET is associated with a frontal-subcortical profile of cognitive impairment. This scale has already been used by other authors for the cognitive assessment of VIM-DBS in patients with ET (12, 27). In addition, it seems to be more sensitive than the MoCA test in the dysexecutive subtype in Parkinson’s disease (PD) patients (28).

#### 2.4.4. Executive functions

Cognitive flexibility will be assessed by the Wisconsin Card Sorting Test, as seen in Fields et al. (12).

Verbal fluency will be measured by timed semantic and phonemic verbal fluency tests (counting the number of words in a minute for each task).

Cognitive inhibition is an executive parameter that has been shown to be impaired in patients with ET and patients with cerebellar pathologies (29, 30). It will be determined by a stroop test (word color) using normative data of the Spanish population for its assessment.

Working memory will be evaluated by the WAIS-IV Working Memory Index. Arithmetic and digit subtests will be used to evaluate processing speed. This test is frequently used to analyse this domain in movement disorders such as PD (31), and it is usually included in the assessment of cerebellar cognitive-affective syndrome (CCAS) (29, 32).

#### 2.4.5. Attention

Attention will be assessed in its different modalities by Trail Making Tests A and B to evaluate selective and divided attention, respectively. These tests have been used by other authors to assess this domain in patients with ET (33) and other movement disorders (31).

#### 2.4.6. Memory and language

Mnesic performance will be assessed with the complutense verbal learning test and logical memory subtests I and II from the Wechsler Memory Scale-IV (WMS-IV). These tests (or their equivalent) are used in other studies on the cognitive assessment of ET (34), VIM-DBS for ET (12), and PD (31). Both tests measure the performance of verbal modality episodic memory, including performance tasks (short- and long-term free recall, with and without cues) and recognition tasks.

Language will be evaluated by the shortened version of the Boston Naming Test, as seen in previous studies on VIM-DBS in ET (12, 27). Along with verbal fluency results, this test offers an accurate and wide picture of language performance as it allows the detection of productive or comprehension deficits.

#### 2.4.7. Visuoperceptual and visuoconstructive function

Visuoperceptual function impairment will be assessed through the Judgement of Line Orientation as seen in other trials (27, 34). Furthermore, our protocol includes the WAIS-IV block-design subtest and the clock-drawing test on command for evaluating these domains after DBS in the different therapeutic targets (once tremor relief has been obtained), although we are aware that other authors use non-motor, visual–spatial, and executive evaluation tasks to avoid tremor artefacting task performance (35, 36).

#### 2.4.8. Quality of life and influence on daily life

The QUEST or Quality of Life in Essential Tremor Scale is included. This is a specific instrument for assessing the quality of life in ET patients, with a validated and normalized Spanish population version.

Moreover, the EuroQol-5D and the Prefrontal Symptoms Inventory (PSI) (37) will also be included in the protocol since the neuropsychological profile of patients with ET can be affected depending on the subjective perception of cognitive performance.

#### 2.4.9. Neuropsychiatric assessment

As depression is one of the mood disorders most consistently associated with ET, the protocol includes Beck’s Depression Inventory-II, as previously used in TE and CCAS (12, 29).

### 2.5. Data analysis

An analysis of cognitive performance in comparison with standard norms will be carried out. The results will be analysed referring to baseline assessments for the different stimulation targets. Numerical variables will be described by absolute and relative frequency measurements. The normality of the variables will be compared using the Shapiro–Wilk test. A student’s t-test will be applied to the paired data to analyse the changes in time of the different parameters within each group. The Wilcoxon test will be used in cases of non-normality. The data will be analysed with R software (R Core Team, 2018. R: A language and environment for statistical computing. R Foundation for Statistical Computing, Vienna, Austria. URL https://www.r-project.org/).

In addition, possible relationships between the results obtained in the neuropsychological variables and the other study variables will be analysed: clinical variables (hemispheric dominance and changes in the tremor), electrical variables (stimulation parameters and volume of tissue activated), and anatomical variables (distance to DRTT obtained through preoperative MRI with dTi). For this, multivariate analyses will be carried out.

## 3. Discussion

Much of the early data on cognitive impairment in ET was brought to light as a result of analysing patients with ET who underwent surgery (thalamotomy or DBS) (12–14, 16). As a result of the spread of surgical treatment for ET, scientific interest in this topic has steadily grown, and the number of studies directed at assessing cognitive impairment in ET has increased exponentially (4). The results to date indicate that patients with ET seem more likely to show a slight overall cognitive decline or depression and anxiety than healthy controls. Furthermore, cognitive deficits in ET are not static and seem to progress faster than in controls (38, 39). Some studies also indicate that patients with ET have a higher risk of dementia (40), and as in other neurodegenerative diseases, the presence of mild cognitive impairment may be a good predictor of future dementia (41).

Most cases manifest cognitive deficits, which usually affect different domains and are generally described as “dysexecutive” or “frontal-subcortical” (42). This has been attributed to cerebellar pathology (4, 36, 43), as cognitive impairment and mood disorders commonly described in patients with ET are similar to those described in CCAS (4). Schmahmann and Sherman outlined this for the first time in 1998 (44), and they also developed and published a targeted diagnostic assessment scale in 2018 (32). They posit that damage at the level of the posterior cerebellar lobe (cognitive cerebellum) or its connections (44, 45) would determine the cognitive changes in CCAS. Thus, the dentate nucleus of the cerebellum is involved in efferences from the posterior cerebellar lobe toward higher-order regions of the cerebral cortex and is considered to participate in cerebellar cognitive processes (45). Therefore, similar neuropsychological results in ET and CCAS support the theory of a cerebellar origin underlying cognitive impairment in ET.

Moreover, patients with ET have more functional difficulties than healthy controls, both in purely cognitive domains and in cognitive-motor domains (46). Cognitive deficits associated with ET are independent of tremor severity, anti-tremor medication, and depression (4, 47); however, there may be an association between cognitive performance and impaired tandem gait (48).

Furthermore, neuropsychiatric impairment is frequently observed in patients with ET, with depression and anxiety being the most frequently observed mood disorders. Other neuropsychiatric disorders described in ET are personality changes, social phobias, and low perceptions of health and quality of life (4). Evidence suggests that depression may be present even before tremor; therefore, the former seems to be independent of motor impairment (49).

Along with executive impairment and tremor severity, neuropsychiatric pathology such as comorbid depression and anxiety has a significant impact on the perception of health and quality of life (QoL), being significantly detrimental to them. However, tremor characteristics such as age of onset, duration, and distribution apparently have no influence on QoL (50, 51).

Several authors have emphasized the need for evidence on both the presence and profile characterization of cognitive-affective impairment in ET patients. Particularly, increased knowledge on the rate of progression and predictors of deterioration, in addition to evidence on how these factors relate to disability and quality of life, could prove beneficial (4, 52, 53).

The potential impact of DBS on cognition in ET patients has seldom been assessed (54). As DBS becomes a standard procedure in ET, the need for data on its clinical efficacy, effectiveness, and potential neuropsychological consequences has become apparent. The potential differences in these spheres depending on the different stimulation targets used are also largely unexplored, especially in more novel anatomical areas such as the PSA.

The development of standardised protocols for neuropsychological assessment in ET surgery is likely to be as useful as in other movement disorders. There is great heterogeneity in the literature regarding this topic, as much in analysed subject populations as in the aims of the studies and battery of tests, which complicates the interpretation and comparison of different studies. In addition, there are significant limitations in the vast majority of studies, such as small sample sizes and the absence of the control group (with a lower average age in the control group when there is one), as well as a lack of correction for confounding factors (4).

In this study, we present a design for a neuropsychological battery and protocol for the investigation of cognitive-affective effects derived from DBS in ET to be applied in subjects that will be stimulated by different anatomical targets, PSA and VIM. Comparison of stimulation at both sites will be possible due to randomization, blinding, and crossing design.

The design of the neuropsychological battery for this study was aimed at covering a wide range of cognitive functions, as described in the previous related literature. Therefore, tests and tasks previously used by other authors for the neuropsychological assessment of ET and other movement disorders (4, 31, 33, 35, 36, 42, 55–57), in studies of DBS for ET (12, 21, 27) and to evaluate CCAS (32) were included. Therefore, if implemented, this proposed neuropsychological assessment protocol might also be extensible to other surgical therapies for ET.

Some authors posit that the detection of executive impairment on ET may be artefacted by the inclusion of tasks that imply intact motor function. Along the same line, rates of non-amnestic cognitive impairment could differ when using a protocol without a motor component (43). To avoid this potential bias, some authors choose neuropsychological evaluations without motor tasks (35, 36, 39). However, Hoche et al. (32) include the block design test on command and as a copy in the CCAS diagnostic assessment scale, arguing that this allows the difficulty of drawing the line on command (“dysmetria of thought”) to be revealed by correcting the copy.

Our proposed protocol tries to minimize the motor component but also includes some tests involving motor tasks, such as the Clock-drawing Test and the WAIS-IV Block-design subtest, allowing executive and visual–spatial functions to be assessed in patients once the motor component has been controlled after DBS.

Finally, the PSA is an area with heterogeneous and complex anatomy, with several adjacent structures that may be involved both in the therapeutic and side effects of DBS. Some studies have tried to assess the function of the PSA and surrounding structures using directional electrodes and VTA. Unfortunately, there were no conclusive results regarding the areas involved in the different side effects, and there was no clear increase in the therapeutic window when using directional electrodes (58).

There are also studies assessing a possible relationship between active contacts-DRTT distance and tremor control, with inconclusive results (59–63). All these findings suggest that more sophisticated and targeted DBS procedures for ET could improve motor and cognitive performance, as well as the side effect profile. In addition, new techniques and technologies such as tractography, VTA analysis, and the creation of probabilistic maps for therapeutic response and side effects may provide extra information from an anatomical and functional perspective.

Nonetheless, few of the studies previously mentioned analyse the relationship between side effects and distance between contacts or VTA and DRTT, and none of them assesses neuropsychological effects to integrate them into this relationship. Our study will aim to correlate the possible neuropsychological findings with the aforementioned anatomical and electrical variables.

### 3.1. Study strengths and limitations

There are several limitations to this study. While it is a wide protocol, this need is derived from the few existing studies and the need to explore a wide range of cognitive functions. Although the protocol will be applied in a single session, to mitigate its duration and avoid patient fatigue, brief breaks will be taken between assessment blocks. Furthermore, the sample size of the clinical trial has been calculated in accordance with the main variable of effectiveness in tremor control but not for identifying neuropsychological changes, so it is possible that the results will not be statistically significant. Finally, although the method of locating the surgical targets is increasingly precise with the advance of neuroimaging techniques, locating these targets will not always be perfect or identical and may cause some unilateral or bilateral losses.

The originality of this study is highlighted as a strength since there are no previous data available related to the cognitive aspects derived from DBS at the PSA level (although there are data on some adjacent structures such as the Zi). Furthermore, due to being a crossover clinical trial, a smaller sample size is required in order to be able to evaluate and compare the effect and allow each patient to act as their own control. This, together with the blinding and random assignment of the stimulation sequence, will ensure good internal validity.

### 3.2. Status of the study

At the time this protocol was published, the recruitment of patients had not been completed. As of today, we have enrolled more than half of the sample, but the follow-up is not complete (completion date expected in mid-2024).

## 4. Conclusion

The present study protocol aimed to evaluate neuropsychological features in a group of ET patients before and after different VIM vs. PSA-DBS surgeries as part of a randomized clinical trial comparing both targets. The results of this study could improve knowledge on the neuropsychological effects of DBS in patients with refractory ET, particularly in lesser-explored targets such as the PSA, as well as being necessary for completing the safety analysis for those procedures. In addition, given the expansion of the indication for surgical procedures in essential tremor, including irreversible ablative procedures, it is important to have an accepted and homogeneous protocol for the neuropsychological evaluation of patients with ET. This proposed neuropsychological assessment protocol could be extensible to other surgical therapies for ET.

## Ethics statement

The study involving humans was approved by the Comité CEIM/CEI Provincial de Granada. The study complies with the requirements established in the Biomedical Research Regulatory Law (Law 14/2007, of 3rd July), will be carried out in accordance with the Declaration of Helsinki 2013 on ethical principles for medical research involving human subjects. The participants will provide their written informed consent to participate in this study.

## Author contributions

LT-C: review of the current status of the topic and wording of the text. BM-R: review of the current status of the topic, and neuropsychological battery selection. CM-N: review of the current status of the topic and study review. MP-N, BI-V, and MK: study review. AM-C: wording of the text and study review. FE-S: review of the current status of the topic, wording of the text, and study review. All authors contributed to the article and approved the submitted version.

## Footnotes

^1^http://www.e-biometria.com
